# Windowed Multitaper Correlation Analysis of Multimodal Brain Monitoring Parameters

**DOI:** 10.1155/2015/124325

**Published:** 2015-03-03

**Authors:** Rupert Faltermeier, Martin A. Proescholdt, Sylvia Bele, Alexander Brawanski

**Affiliations:** Department of Neurosurgery, University Hospital Regensburg, Franz-Josef-Strauß-Allee 11, 93042 Regensburg, Germany

## Abstract

Although multimodal monitoring sets the standard in daily practice of neurocritical care, problem-oriented analysis tools to interpret the huge amount of data are lacking. Recently a mathematical model was presented that simulates the cerebral perfusion and oxygen supply in case of a severe head trauma, predicting the appearance of distinct correlations between arterial blood pressure and intracranial pressure. In this study we present a set of mathematical tools that reliably detect the predicted correlations in data recorded at a neurocritical care unit. The time resolved correlations will be identified by a windowing technique combined with Fourier-based coherence calculations. The phasing of the data is detected by means of Hilbert phase difference within the above mentioned windows. A statistical testing method is introduced that allows tuning the parameters of the windowing method in such a way that a predefined accuracy is reached. With this method the data of fifteen patients were examined in which we found the predicted correlation in each patient. Additionally it could be shown that the occurrence of a distinct correlation parameter, called scp, represents a predictive value of high quality for the patients outcome.

## 1. Introduction

The outcome of patients suffering from catastrophic neurological events such as traumatic brain injury (TBI) or subarachnoid hemorrhage (SAH) is influenced by two types of pathophysiological mechanisms: the primary injury sustained at the moment of impact and the secondary injury caused by a cascade of ischemic, ionic, and neurochemical insults which evolve over time after the primary insult [1]. Since the primary injuries are merely irreversible, the main focus of neurocritical care is to detect and prevent secondary brain injury [2]. Consequently, an array of different monitoring techniques for the assessment of intracranial pressure (ICP), oxygenation status, and metabolism has been developed to unmask occult local and systemic alterations of the brain [3–6]. However, the resulting high volume of multimodal datasets frequently exceeds the ability of the neurointensivist to process and integrate these data into an adequate treatment algorithm [7]. There is an urgent need for the development of advanced biomathematical tools to analyze the complex and interwoven data sets in real time guiding a goal directed therapy of these patients [8]. Our group has recently developed a mathematical model which allows simulating brain swelling and loss of cerebral autoregulation [9]. In case of a severe brain swelling, this model predicts a long-term positive correlation between arterial blood pressure (ABP) and ICP if the cerebral autoregulation is experimentally switched off, in contrast to a negative correlation for an intact autoregulation [10]. The main goal of our study was to develop a set of mathematical tools that reliably identify such positive and negative correlations in bimodal monitoring data. The correlations are described by three classification numbers, called selected correlation (sc), selected correlation positive (scp), and selected correlation negative (scn). The sc index is deduced from windowed multitaper coherence calculations and multitaper power spectra [11]. The phasing of the data windows is detected by the mean value of the windowed Hilbert phase difference [12] leading to the indices scp and scn. For all indices a statistical testing method is presented to determine the significance of the particular results. In a second step we calculated the percentage allocations of positive and negative correlations and related the results to the outcome of the analyzed patients. We hypothesized that patients showing predominantly positive correlations will have a poor prognosis. In addition we attempt to validate the predicted episodes of diminished cerebral compliance by analysis of CT scans.

## 2. Methods

### 2.1. Patient Population

The study was conducted in accordance with the ethical guidelines of the University Regensburg Institutional Review Board. Informed consent was obtained from the patient's relatives, all study results were stored and analyzed in an anonymized fashion. We prospectively investigated a cohort of 15 adult patients (5 female, 10 male) who were treated at the neurosurgical intensive care unit for traumatic brain injury (TBI) or subarachnoid hemorrhage in 10 and 5 cases, respectively. The mean age was 43.7 years (range: 20.2–72.4), the median Glasgow Coma Scale GCS at the time of admission was 7 (range: 3–15). The mean duration of multimodal monitoring was 142 hours (range: 20.3–349.4). The detailed patient characteristics are summarized in Table 1. All patients were sedated and mechanically ventilated during the observation period and received an intra-arterial catheter for the continuous measurement of arterial blood pressure as part of the standard treatment procedure in our institution. ICP monitoring was performed continuously using either an external ventricular drain equipped with an electronic pressure device (EVD) or a parenchymal ICP probe (both from Raumedic, Helmbrechts, Germany). The ABP and ICP data were acquired continuously using a data logger (Daq USB 6210, National Instruments, Munich, Germany) with a sample frequency of 1000 Hz. For the correlation analysis, the data were resampled to 0.2 Hz (one data point every five seconds) to reduce noise effects and to smooth out fast oscillations or spikes. Computed tomography imaging was conducted when clinically indicated. Followup was completed up to July 2013 by reviewing outpatient records and contacting the patients family member or the patient's primary physician. The mean followup time was 77.9 months, no patient was lost for followup. The neurological outcome was measured by the Glasgow Outcome Scale (GOS) [13], and revealed a median score of 3 (range: 1–5) at the final followup exam.

### 2.2. Correlation Index Calculations

To identify the above mentioned positive and negative correlations between ABP and ICP in monitoring data from the ICU, we used a Fourier based method, as described in detail previously [14]. In brief, we adopt coherence and power spectra calculations with the multitaper method (MTM) to determine the coherence between segments of two time series that were synchronously recorded with a sampling rate of 0.2 Hz. Additionally, we calculate the mean Hilbert phase difference between these segments to determine the phasing of the correlation. The MTM [11, 15], based on fast Fourier transforms (FFT), is a sophisticated way to determine power spectra and coherence of data using a set of so-called Slepian tapers as window functions to handle noisy and short data. Additionally the MTM software comes with built-in statistical tests proving the significance of each frequency. The principle approach of the above mentioned windowed MTM calculations can be described as follows: From the isochronous time series *A*, *I* select windows *w*_*k*_^*A*^, *w*_*l*_^*I*^ of fixed size *s* with *s* = 2^*z*^, *z* ∈ ℕ and potentially different starting points *k* and *l*. For this pair of windows we calculate the mtm-spectra mtms(*f*_*i*_) and the mtm-coherence between the windows (mtmc(*f*_*i*_)):

(1) $$\begin{array}{c} w_{k}^{A}≔\left(x_{k},\ldots ,x_{k+s-1}\right),\\ w_{l}^{I}≔\left(y_{l},\ldots ,x_{l+s-1}\right),\\ S_{l}^{I}\left(f_{i}\right)≔\text{mtms}\left(f_{i}\right)\;\;\text{of}\;w_{l}^{I},\\ C_{k,l}^{A,I}\left(f_{i}\right)≔\text{mtmc}\left(f_{i}\right)\;\;\text{of}\;w_{k}^{A},w_{l}^{I}. \end{array}$$

With the above mentioned setting for the window size *s* the MTM-spectra and MTM-coherence contain exactly *s*/2 frequencies *f*_*i*_. Using the built-in significance test of the MTM each single frequency *f*_*i*_ is tested for significance. Thus, we can define the pointwise selected correlation (PSC) assuming a significance level for the built-in significance test of 99%:

(2) PSCk,lA,I≔psc1A,Ik,l,…,pscs/2A,Ik,l  with:psciA,Ik,l=1if  SkAfi,SlIfi,Ck,lA,Ifisig.0otherwise.

The PSC represents a list of length *s*/2 with elements of value 1 if the correspondent frequency is considered significant in the spectra and the coherence; otherwise the value of the element is 0. The significance of *f*_*i*_ in both spectra guarantees that only frequencies are considered that essentially contribute to the original signals, whereas the significance in the coherence assures that a specific *f*_*i*_ implies correlation between the input signals. Repeating the PSC calculations for *N* pairs of isochronous windows leads to the mean pointwise selected correlation (MPSC):

(3) $$\begin{array}{c} {\text{MPSC}}^{A,I}=\overset{j=N}{\underset{j=1}{\sum }}{\text{PSC}}^{A,I}\left(j,j\right). \end{array}$$

The elements of the MPSC list represent the percentage of being significant in both spectra as well as in the coherence calculation for each single frequency *f*_*i*_. Therefore, using MPSC we are able to determine frequency intervals reflecting relevant correlations within a whole data set. If distinctive correlations occur in the data set, the related frequencies *f*_*k*_ lead to an increase in the index MPSC_*k*_(*f*_*k*_). Calculating the MPSC for a complete data set reveals in which frequency range correlations occur on average. After having identified such a frequency interval *U* = (*f*_*m*_,…, *f*_*n*_) we want to determine periods in the data set where strong correlation with respect to *U* occurs. To do so, we first estimate the degree of correlation of a distinct pair of windows with respect to the interval *U* by calculating the sum of all elements psc_*k*_ of PSC belonging to the frequencies in *U*. This sum divided by the length of *U* is called selected correlation (sc):

(4) scm,nA,Ik,l≔1n−m+1∑j=mj=npscjA,Ik,lhhhhhhhhh0≤m<n≤s2.

**Figure 1:**
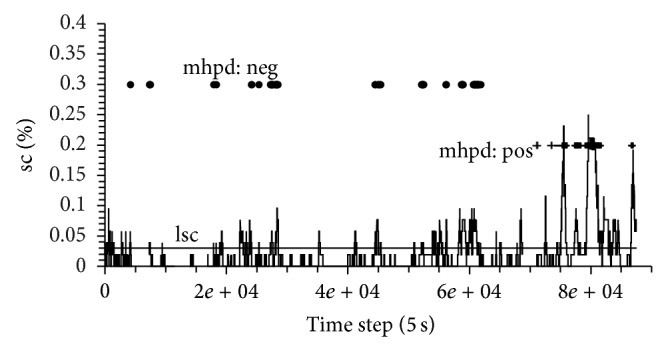
Time evolution of the sc value of a patient included in this study. Each time the sc value is higher than limit lsc a profound correlation between ABP and ICP is detected. If additionally the mhpd value surpasses either lmhpd_pos_ or lmhpd_neg_ a distinctive negative correlation (depicted as dots) or a distinctive positive correlation (depicted as crosses) is detected.

A pair of windows is called selected correlated if sc > lsc for any chosen threshold lsc. A statistical test, to be described later on, is used to determine the significance of the lsc value. The sc value therefore serves as a measure for the degree of correlation of a pair of data windows with respect to a specific frequency range where the specified correlation occurs. Consequently, to gather time resolved information about the correlation between ABP and ICP time series in a specific frequency range, we determine the index sc_*m*,*n*_^*A*,*I*^ (*l*, *l*) for isochronous windows while shifting the starting point *l* along the time axis (Figure 1).

### 2.3. Hilbert Phase Differences

Having identified a pair of windows exhibiting a sufficient high correlation index sc, we calculate, in a next step, the Hilbert phase difference [12] of the corresponding time series segments to detect the phasing of the signals. For two time series *a*(*t*), *b*(*t*) the Hilbert phase difference is defined by the components of the corresponding analytical signals,

(5) aanalytic=at+j∗a~t=At∗ej∗φt  with:at=π−1∗P.V.∫−∞∞a(τ)t−τdτhpdt=arctan⁡a~tbt−atb~tatbt−a~tb~t,

where P.V. stands for the Cauchy principal value of the of the integral considered. Prior to this calculation both segments are normalized to zero mean and unit standard deviation, just as it is done for the MTM-calculations. From the resulting hpd(*t*) we calculate the mean value of the Hilbert phase difference as a measure for the phasing of the two input windows. A correlation is called negative if the mean Hilbert phase difference (mhpd) is higher a predefined limit lmhpd_neg_ and correspondingly is called positive if lower a predefined lmhpd_pos_. Finally, a statistical test is used to determine the significance of lmhpd_neg_ and lmhpd_pos_.

### 2.4. Statistical Test

The statistical test, presented in the following, is a specific type of perturbation test ([16]) and based on the model prediction that correlations between ABP and ICP occur isochronous. Consequently, two segments should not be correlated if their starting points are quite apart from each other and the corresponding sc value should be zero or at least very low. Assuming that a sc value is meaningful if it is higher a predefined specific threshold lsc, we can count how often this separated and therefore uncorrelated windows produce sc values higher than lsc. From this obviously wrong hit we deduce the error rate and finally the significance of the sc value with respect to the predefined threshold. In a first step we will determine an appropriate separation length, or time shift, to guarantee the uncoupling of the time series with respect to the chosen segment length *s*. If at least one of the signals exhibits a distinctive autocorrelation the time shift between the segments has to be chosen big enough to minimize this source of irritation. First we construct test data sets ABP_all_ and ICP_all_ by simply joining the appropriate data sets of all patients involved. Within such a test data set we randomly determine a starting point for the first window of length *s*. The starting point of the second window, then, results from the first starting point plus a specified time shift *o*. Subsequently we can calculate the sc value for this distinct pair of windows. To cover the characteristics of the whole test data set this procedure is repeated *E* times. Afterwards, the time shift *o* is increased, and the whole procedure is repeated until ending in the calculation of the mean windowed autocorrelation (mwa):

(6) $$\begin{array}{c} {\text{mwa}}^{A}\left(o\right):=\left(\frac{1}{E}\right)\overset{e=E}{\underset{e=1}{\sum }}{\text{sc}}_{m,n}^{A,A}\left(e,e+o\right). \end{array}$$

If the time shift *o* if large enough to exclude autocorrelation artifacts, the subsequent mwa values should be small and stable. With the ability to exclude interference from autocorrelation effects, the above mentioned calculation of the error rate for sc with respect to a predefined threshold lsc could be done as follows: Using the ABP_all_ and ICP_all_ test data sets for the generation of the input windows, we repeatedly and randomly determine pairs of starting points (*a*_*i*_, *b*_*i*_) with time shift much bigger than autocorrelation effects (|*a*_*i*_ − *b*_*i*_| > *o*_ac_). Again this is done to cover the characteristics of the whole test data set. For the associated pairs of nonisochronous windows, we calculate the selected correlation sc_*m*,*n*_^*A*,*I*^ (*a*_*i*_, *b*_*i*_). As correlations should only occur for isochronous windows these values of sc reflect a threshold that should be passed by correlated windows. In other words, if we call a sc value meaningful if higher than a predefined threshold lsc, all sc values of such nonisochronous windows satisfying the lsc criterion are wrong hits. Accordingly, we deduce the error index ei_*m*,*n*_^*A*,*I*^ (*a*, *b*), indicating whether the selected correlation sc_*m*,*n*_^*A*,*I*^ (*a*, *b*) is higher than a predefined limit lsc and the error rate asc, that is, the rate of obviously wrong hits with respect to lsc:

(7) $$\begin{array}{c} {\text{ei}}_{m,n,\text{lsc}}^{A,I}\left(a,b\right)≔\left\{\begin{array}{cc} 1 & \text{if}\;{\text{sc}}_{m,n}^{A,I}\left(a,b\right)>\text{lsc}\\ 0 & \text{otherwise}, \end{array}\right.\\ {\text{asc}}_{m,n}^{A,I}\left(\text{lsc}\right):=\left(\frac{1}{K}\right)\overset{i=K}{\underset{i=1}{\sum }}{\text{ei}}_{m,n,\text{lsc}}^{A,I}\left(a_{i},b_{i}\right). \end{array}$$

The resulting error rate asc describes the percentage of obviously wrong hits in the above mentioned sense with respect to a predefined limit lsc. Thus asc represents the probability of a distinct value sc > lsc to be generated by accident and therefore describes the significance of sc with respect to lsc. The above described method can easily be modified to calculate the error rates of the mean Hilbert phase difference (mhpd) by substituting the lsc criterion in ei_*m*,*n*_^*A*,*I*^(*a*, *b*) through appropriate criteria lmhpd_pos_ and lmhpd_neg_. If mhpd > lmhpd_neg_ the correlation between the appropriate windows will be called negative; if mhpd < lmhpd_pos_ the correlation will be called positive. Accordingly, the error rates will be named amhpd_pos_ and amhpd_neg_, respectively, and will be used to determine the significance of the lmhpd_pos_ and lmhpd_neg_. The above described tools will be used to determine for an arbitrary pair of input windows whether these windows are significantly correlated and, if so, whether the correlation is significantly positive or negative. Shifting the input windows synchronously along the time axis of the measured data produces a time resolved information about the different possible correlations (see Figure 1). Additionally we will calculate the percentage of correlated windows with respect to lsc and the percentage of distinct positive and negative correlations, with respect to lmhpd_pos_ and lmhpd_neg_. The percentage of distinct positive correlated windows will be termed as selected correlation positive (scp) and correspondingly the percentage of distinct negative correlated windows will be termed as selected correlation negative (scn).

## 3. Results

**Figure 2:**
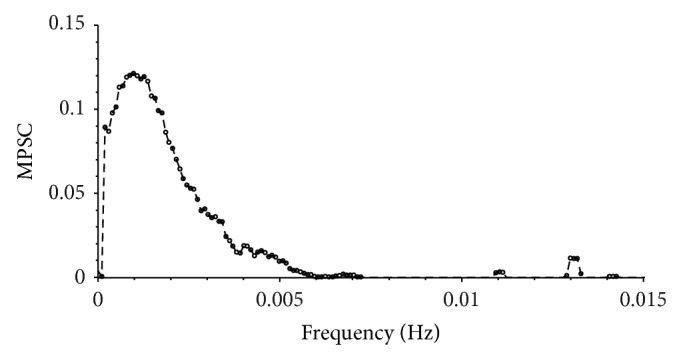
Mean pointwise selected correlation (MPSC) of ABP signal of all included patients. After reaching its maximum at about *f* = 0.001 Hz a sharp decrease follows. For frequencies *f* > 0.005 Hz the MPSC values almost reach zero apart from a few local maxima that are far less pronounced than the low frequency band.

**Figure 3:**
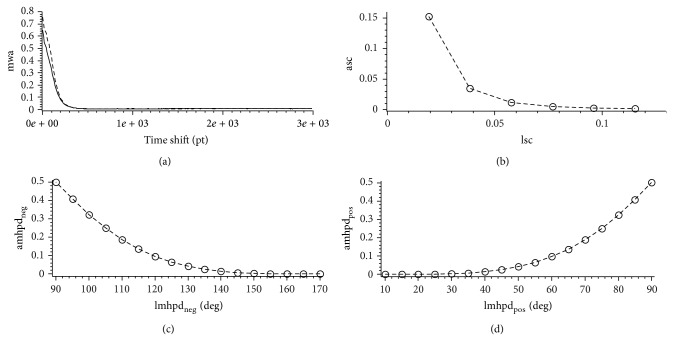
Mean windowed autocorrelation (mwa) of the ABP (dashed line) and ICP (solid line) signal for a window size of 2048 points (a). The mwa value decreases very rapidly for both signals. For offsets greater than 500 data points the mwa value remains stable at mwa < 0.011 (a). Error rate asc with respect to sc: choosing the criterion for a pair of windows to be correlated as lsc = 0.03 leads to a asc < 0.05 (b). Error rate amhpd_neg_ with respect to the limit lmhpd_neg_ for correlations to be called negative: a lmhpd_neg_ of 130 deg for the mean Hilbert phase difference (mhpd) generates an amhpd_neg_ < 0.05 (c). Error rate amhpd_pos_ with respect to the limit lmhpd_pos_ for correlations to be called positive: a lmhpd_pos_ of 50 deg for the mean Hilbert phase difference generates an amhpd_neg_ < 0.05 (d).

**Figure 4:**
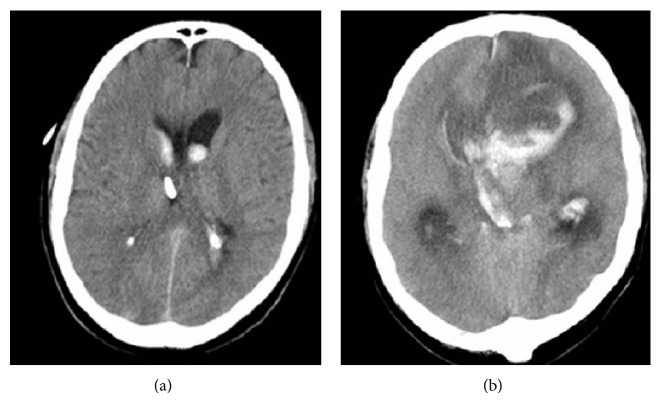
Serial CT scans of a patient following SAH (GCS 8). Initial scans show an intraventricular hemorrhage without signs of stroke or brain edema (a). Followup scan performed at a period of selected positive correlation between ABP and ICP shows severe brain edema reducing the subarachnoid space, a diminished demarcation of the cortical-subcortical border, and a partial stroke of the anterior cerebral artery (b).

For all subsequent calculation a window size *s* of 2048 data points was used. In Figure 2 the MPSC value for each individual frequency is depicted. For this analysis the time series of all included patients were used. The MPSC value reaches its maximum of about 0.12 at a frequency of approximately 0.001 Hz and then rapidly decreases. Between 0.005 Hz and 0.008 Hz MPSC values reach their lower limit of 0.0. Additional bands appear at 0.011 Hz and 0.013 Hz which are far less distinct. For a better visualization of the lower frequency band Figure 2 only shows the MPSC value for frequencies lower than 0.015 Hz. For frequencies higher than 0.015 Hz MPSC values are mostly zero except of a view small regions with MPSC values lower than 0.00012. Therefore we choose a frequency interval of *f* < 0.005 Hz for further analysis. The mean windowed autocorrelations (mwa) of ABP signal and ICP signal are depicted in Figure 3(a), where 5000 different pairs of input windows were used for the calculation of a single offset *o*. If no time shift is applied (step size 0), the mwa value is highest but not one. This is due to the fact that in this case all exploited frequencies are sufficiently correlated but some of them prove to be not significant in the spectra. With growing step size the mean windowed autocorrelation decreases rapidly for both signals and at least after a step size of half the window size is reached and mwa is low and stable (mwa < 0.011). From this we can conclude that if the windows are simply nonoverlapping, the time shift is big enough to exclude autocorrelation effects in both signals. Therefore we used an offset *o* of three times the window size (*o* = 3∗*s*) for the statistical testing. For the calculation of the different error rates 2 × 10^6^ different pairs of input windows were used. The resulting error rates asc, amhpd_pos_, and amhpd_neg_ are depicted in Figures 3(b)–3(d). From lsc > 0.03 an asc < 0.05 follows. If we choose lsc > 0.1, asc reduces to asc < 0.0015. Choosing the upper and lower limits for the mean Hilbert phase difference according to earlier work from our group [10] as 130 deg and 50 deg results in amhpd_neg_, amhpd_pos_ < 0.05. Using 150 deg a 30 deg for the upper and lower limit reduces the error rates, amhpd_pos_, amhpd_neg_ < 0.004 (see Figures 3(c) and 3(d)). To achieve a significance level higher than 95% for the detection of correlations we therefore used lsc = 0.03 and lmhpd_neg_ = 130 deg, lmhpd_pos_ = 50 deg for the detection of negative and positive correlations. Using the above defined thresholds, significant correlations between the parameters ABP and ICP were recorded in all patients; however, the time span of correlating parameters per observation period differed considerably between the individual patients (see Table 1). All patients, from which CT scans were obtained during positive selected correlation phases, indicating a loss of autoregulation combined with reduced intracranial compliance, showed dramatic changes indicating severe edema, hemorrhagic transformation, or brain ischemia (Figure 4).

**Figure 5:**
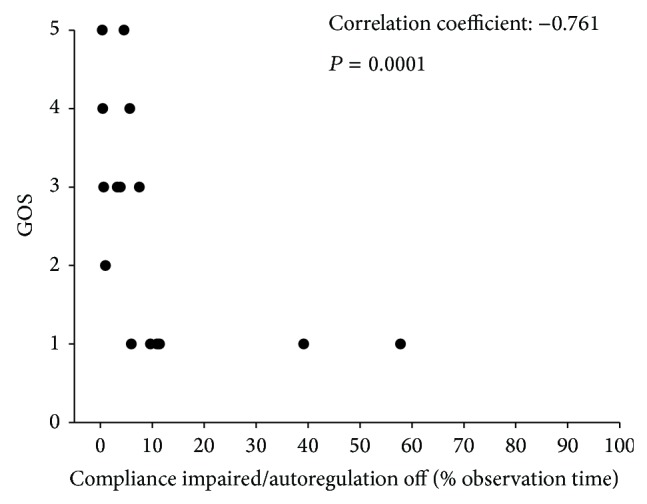
Relation between the selected correlation positive (scp) and the outcome measured by the Glasgow Outcome Score. Spearman correlation analysis revealed a highly significant association between the parameters (*P* < 0.0001). Note that a scp greater than 10% of the observation time resulted in the death of all afflicted patients.

In addition, the value of selected correlation positive (scp) during the observational period correlated highly significantly with the patients outcome as measured by the Glasgow Outcome Scale (*P* < 0.0001, see Figure 5). Interestingly, all patients showing scp values higher than 10% died during the course of the disease. Multiple regression analysis including age, diagnosis, scp, and the GCS score at admission revealed the selected correlation positive as independent factor for patients outcome (*P* < 0.001).

## 4. Discussion

Despite promising in vivo results in several animal models, investigating neuroprotective agents such as glutamate antagonists, free radical scavengers, or growth factors, clinical trials have failed to show any benefit for patients with TBI or SAH [17, 18]. Since causal treatment is lacking so far, the main goal of neurointensive care is to provide an optimal physiological and cellular environment to prevent secondary injury and to facilitate endogenous regeneration of the injured brain [19]. To achieve this mission, a number of technically sophisticated monitoring devices measuring ICP [20], brain oxygenation [21], metabolism [22], cerebral blood flow [23], and EEG activity [24] have been developed recently. However, the interpretation of these multimodal data sets is until today based on experience and adequate intuition of the treating neurointensivist, who may be facing more than 200 variables per patient [6]. As shown previously, it is almost impossible to grasp the interrelation between more than two data sets without the support of biomathematical analysis tools [25]. For example, an elevated ICP may require the increase of the systemic blood pressure in a patient with functional autoregulation to provide adequate cerebral perfusion pressure (CPP), whereas it can be harmful to a patient with a loss of autoregulation [2]. It is therefore necessary to avoid dogmatic threshold values as a foundation for treatment algorithms [26] but to individually tailor therapeutic intervention for each patient [5, 7]. This aspect becomes even more vital as recent studies have demonstrated significant adverse effects of treatment strategies targeting CPP or brain oxygenation if executed inadequately [27, 28]. The main goal of our study was therefore to employ advanced biomathematical tools to identify patients at risk and to improve the pattern of care and prognostication in patients treated in the neurointensive care unit. With only 15 patients, the sample size of our cohort is rather small, which may be a potential limitation. Further studies are underway to prospectively validate our findings and their potential impact on treatment outcome.

In conclusion, the used methods are able to reliably detect correlations with different phasing as predicted by a mathematical model. By means of a statistical testing method the accuracy of the results could be tuned as needed for a reliable judgment of the patients status. Interestingly, the frequency of positive selected correlations highly was identified as an independent factor for the prognosis of our study patients. Our results indicate that a real time application of this analysis would potentially allow for an early intervention improving the outcome of patients by preventing secondary injury in a more timely fashion [29].

## Figures and Tables

**Table 1:** Characteristics of patients included in the study who were treated for either SAH (subarachnoid hemorrhage) or TBI (traumatic brain injury). The column GCS describes the level of consciousness at admission according to the Glasgow Coma Scale. Measuring time reports the duration of multimodal monitoring interval per patient. Over all correlation describes the percentage of significant correlations within the observation period. The last column indicates the outcome determined by the Glasgow Outcome Scale at the final follow up.

ID	Gender	Age	Diagnosis	GCS	Measuring time (hours)	Overall Correlation (%)	GOS
1	m	54.8	SAH	6	173.87	4.45	1
2	m	51.6	SAH	8	118.16	48.74	1
3	f	26.2	TBI	7	20.28	75.91	5
4	f	20.2	TBI	3	68.09	83.17	1
5	m	53.0	SAH	3	191.67	72.65	1
6	m	42.6	SAH	15	316.41	30.06	3
7	f	60.2	TBI	10	349.37	50.90	3
8	f	72.4	TBI	14	241.86	32.94	3
9	m	21.5	TBI	7	53.4	30.97	2
10	m	42.7	TBI	11	123.26	22.77	1
11	m	51.7	TBI	3	140.74	24.60	3
12	m	33.1	TBI	3	123.1	7.91	4
13	m	49.9	TBI	14	40.29	16.74	1
14	f	42.8	TBI	5	95.58	21.46	4
15	m	32.7	TBI	10	78.66	7.86	5

## Abbreviations

amhpd_neg_: Error rate for limit of negative mhpd (percentage of false positives of mhpd with respect to lmhpd_neg_)
amhpd_pos_: Error rate for limit of positive mhpd (percentage of false positives of mhpd with respect to lmhpd_pos_)
asc: Error rate of selected correlation (percentage of false positives of sc with respect to lsc)
ei: Error Index (ei is defined as 1 if the sc value is higher than lsc and 0 otherwise; for data windows of abp and icp with an offset, ei indicates false positives)
FFT: Fast Fourier transform (method to calculate the discrete Fourier transformation of a dataset)
hpd: Hilbert phase difference (difference of the Hilbert phase of two signals)
lmhpd_neg_: Limit mean Hilbert phase difference negative (arbitrary limit for mhpd to indicate a negative phasing)
lmhpd_pos_: Limit mean Hilbert phase difference positive (arbitrary limit for mhpd to indicate a positive phasing)
lsc: Limit selected correlation (arbitrary limit for sc to indicate correlation)
mhpd: Mean Hilbert phase difference (mean value of the Hilbert phase difference for a fixed data window)
MPSC: Mean pointwise selected correlation (element by element mean of PSC vectors of a selection of data windows)
MTM: Multitaper method (sophisticated fft based method to calculate power spectra and coherence from a data window)
mtmc: Multitaper coherence (coherence calculated with multitaper method)
mtms: Multitaper spectrum (power spectrum calculated with multitaper method)
mwa: Mean windowed autocorrelation (mean value of sc for data windows of identical data (abp or icp) with a predefined offset but varying starting points)
PSC: Pointwise selected correlation (a vector containing a 1 if the corresponding frequency is significant in the spectra and the coherence and 0 otherwise; the PSC is calculated for a fixed pair of corresponding data windows)
sc: Selected correlation (sum over the elements of PSC normed by the length of PSC)
scn: Selected correlation negative (percentage of sc with additionally a negative mhpd)
scp: Selected correlation positive (percentage of sc with additionally a positive mhpd).
